# Dynamic Behavior of Nanocomposites Reinforced with Multi-Walled Carbon Nanotubes (MWCNTs)

**DOI:** 10.3390/ma6062274

**Published:** 2013-06-03

**Authors:** Shiuh-Chuan Her, Chun-Yu Lai

**Affiliations:** Department of Mechanical Engineering, Yuan Ze University, 135 Yuan-Tung Road, Chung-Li 320, Taiwan; E-Mail: s975028@mail.yzu.edu.tw

**Keywords:** multi-walled carbon nanotube, functionalization, natural frequency, damping ratio

## Abstract

The influence of multi-walled carbon nanotubes (MWCNT) on the structural dynamic behavior of MWCNT/epoxy nanocomposites was investigated. Two different types of MWCNTs, pristine MWCNT and functionalized MWCNT, were used in this study. Carboxylic acid-functionalized MWCNTs (MWCNT-COOH) were obtained by oxidation pristine MWCNTs via sonication in sulfuric-nitric acid and characterized by Fourier transform infrared spectroscopy (FTIR). Dynamic behaviors of the MWCNT reinforced nanocomposite including the natural frequency and damping ratio were determined using free vibration test. Experimental results showed that the damping ratio of the nanocomposite decreases with the increase of the MWCNT addition, while the natural frequency is increasing with the increase of the MWCNT addition. Functionalized MWCNTs improved the interfacial bonding between the nanotubes and epoxy resin resulting in the reduction of the interfacial energy dissipation ability and enhancement of the stiffness.

## 1. Introduction

The increasing demand for high-quality and multifunctional materials in different industrial applications have promoted huge research effort on the formulation and preparation of advanced nanostructured composites with superior characteristics. Carbon nanotubes (CNTs) exhibit a wide range of excellent mechanical, optical, and electrical properties along with chemical stability. Their mechanical properties, such as Young’s modulus and tensile strength, considerably exceed those of currently available materials [1]. With excellent mechanical, electrical and thermal properties, CNTs are considered to be ideal for reinforcing high performance composites and enable the fabrication of composites with unique multi-functional characteristics. Recent research articles have focused on the use of CNTs in polymer matrix composites. Different polymer/CNT nanocomposites have been prepared by incorporating CNTs into various polymer matrices, such as polyamides [2], polyimides [3], epoxy [4], polyurethane [5] and polypropylene [6]. Among these various polymer matrices, epoxy resin is one of the most often used polymer matrix for advanced composite applications in automotive, aeronautics, marine industry, electronics and others. The group of resins of this family presents good stiffness and specific strength, dimensional stability, chemical resistance, and also strong adhesion to the embedded reinforcement [7]. The preparation of CNT-reinforced epoxies and any other kind of polymer, however, requires a homogeneous dispersion and a strong interfacial interaction between the nanotubes and the polymer [8]. However, high specific surface areas of CNTs cause a strong tendency to agglomerate, which reduces the strength of the nanocomposites as the results of inhomogeneous dispersion and stress concentration in the polymer matrix [9]. The practical uses of pristine CNTs are limited by the tendency of agglomeration and insolubility in organic solvents or water. Many researchers have proposed a variety of methods to overcome barriers to prepare CNTs-reinforced nanocomposites, and the most effective method is chemical functionalization [10,11,12,13,14], which could provide functional groups on the surface of CNTs and form covalent bonding with matrix further. However, strong acid treatment would cut off CNTs’ length and limit its application as high performance filler [15,16,17]. The functionalization of CNT is an effective way to prevent nanotube aggregation, which helps to better disperse and stabilize the CNTs within a polymer matrix. Ma *et al.* [18] reported that grafting silane molecules onto the CNT surface improved the dispersion of CNTs in epoxy along with much enhanced mechanical and thermal properties as well as fracture resistance of nanocomposites compared to those containing CNTs without functionalization. Lavorgna *et al.* [19] investigated the effects of Silanization and silica enrichment of multi-walled carbon nanotubes on the thermal-mechanical properties of epoxy nanocomposites. They found that in the rubbery region, the storage modulus of composites with silanized and silica enriched carbon nanotubes were about 240% and 285% higher than modulus of neat epoxy system, respectively. Most studies on polymeric composites with functionalized CNTs focus on elastic properties. Relatively little attention has been given to their damping mechanisms and ability. Few have investigated the effect of nanotube functionalization on damping properties of polymeric composites [20]. Kordani *et al.* [21] investigated the structural damping characteristics of polymeric composites containing carbon nanotubes with various kinds and amounts. Finegan *et al.* [22] studied the mechanical damping and storage modulus of carbon nanofiber/polypropylene composites using a Dynamic Mechanical Thermal Analyzer. Zhou *et al.* [23] proposed a model of interfacial “stick-slip” frictional motion between the nanotubes and the resin to examine the damping properties of CNT-based composites. Savvas *et al.* [24] presented a multiscale model to investigate the effect of interfacial shear strength on the mechanical and damping properties of carbon nanotube reinforced composites. Liu *et al.* [25] studied the effect of functionalization of single-wall carbon nanotubes on the damping characteristics of SWNT-based epoxy composites. From the applications point of view, vibration damping forms an important aspect of structural health monitoring. Several military equipment, automobiles, aircrafts and spacecrafts presently suffer from the menace of vibrations [26]. Microcracks present in these structures propagate rapidly due to fatigue loading caused by vibrations, resulting into their catastrophic failure. Koratkar *et al.* [27,28] observed promising damping ability of a densely packed MWNT thin film (no matrix); however, damping characteristics of CNT filled composites have not been investigated in any detail.

In this work, MWCNTs were dispersed in a mixture of sulfuric and nitric acid. Carboxylic acid-functionalized MWCNT (MWCNT-COOH) were obtained by oxidation of pristine MWCNTs. The effect of functionalization on the damping ratio and natural frequency of MWCNT reinforced nanocomposites was investigated. Free vibration test was conducted to determine the damping ratio and natural frequency of the nanocomposites.

## 2. Preparation of MWCNTs reinforced nanocomposites

### 2.1. Materials

The polymer matrix consisted of part A epoxy resin (Bisphenol A diglycidyl ether C_21_H_24_O_4_) and part B hardener (Tetraethylenepentamine 80%, Fatty Acid 20%), both purchased from Glad Co. Taiwan. Two different types of MWCNTs, pristine MWCNT and functionalized MWCNT, were used in this work. Pristine multi-walled carbon nanotubes grown by chemical vapor deposition method were purchased from Golden Innovation Business Co. and its purity was higher than 95%. The MWCNTs had diameter ranging from 40 to 60 nm, and the length ranging from 1 to 5 µm. Distilled water was used in all procedures if necessary.

### 2.2. Functionalization of MWCNTs

In order to obtain uniform dispersion and strong interactions between the carbon nanotubes and the epoxy matrix, carboxylic acid functional groups were grafted onto the nanotube surface. This synthetic functionalization procedure has been reported and briefly described as follows. MWCNTs were dispersed in a mixture of sulfuric and nitric acid with volume ratio of 3:1. The MWCNTs in solution was sonicated in an ultransonic bath for 6 h at ambient temperature. Carboxylic acid-functionalized MWCNTs (MWCNT-COOH) were obtained by oxidation pristine MWCNTs via sonication in sulfuric-nitric acid. After sonication, the solution was diluted with large amount of distilled water. The functionalized MWCNTs were filtered through a membrane with pore size of 0.45 µm and repeatedly washed with distilled water. The concentration of acidity was measured periodically. The MWCNTs were considered to be acid free when the pH of MWCNTs in the solution is equal to the pH in the distilled water. The functionalized MWCNTs were then dried in a vacuum oven at temperature of 80 °C for 24 h and denotes as MWCNT-COOH.

### 2.3. MWCNT/Epoxy Nanocomposites

**Figure 1:**
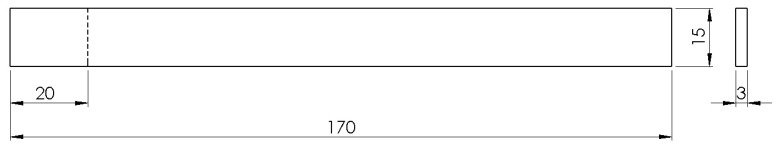
Free vibration testing specimen (unit: mm).

A desired amount of MWCNTs was directly added into a liquid epoxy which was preheated at temperature of 60 °C for 30 min. The solution was sonicated in an ultransonic bath for 3 h at temperature of 50 °C to separate the aggregation of the MWCNTs and achieve good dispersion. The solution was degassed in a vacuum oven maintained at pressure of 20 mm Hg for 3 h. Then, the epoxy hardener AH150 was mixed into the MWCNT/epoxy solution, and softly stirred it for about 10 min. The mixing of epoxy and curing agent initially produced highly reactive, volatile vapor bubbles, which could create voids and significantly affect the properties of the final product. To reduce the chance of voids, the liquid was preheated to 50 °C to reduce its viscosity. Then, the solution was placed in a vacuum chamber for about 30 min to remove the bubbles induced from the stirring. After the bubbles were completely removed, the nanocomposite suspension was poured into the aluminum mould to fabricate the test specimen as shown in Figure 1 with the dimension of 170 mm (length) × 15 mm (width) × 3 mm (thickness). All the specimens were post-cured in a vacuum oven at temperature of 50 °C for 24 h. Samples without the MWCNT addition were also fabricated for comparison. Prior to the free vibration test, the specimen surfaces were mechanically polished to reduce the influence of surface flaws. Specimens of neat epoxy and MWCNT/epoxy nanocomposites with two different kinds of MWCNTs, *i.e.*, pristine MWCNTs and functionalized MWCNT-COOH were prepared in the same procedures. The contents of the MWCNTs are ranging from 0.3 to 1.0 wt % in this work.

## 3. Characterization

**Figure 2:**
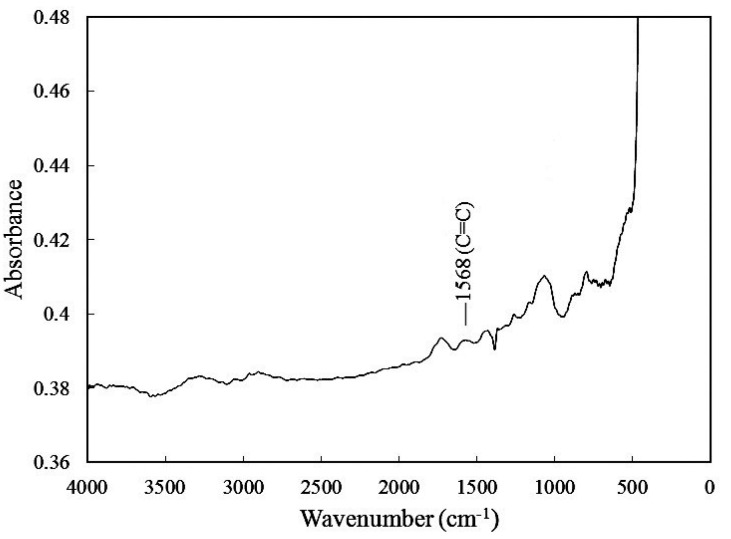
Fourier transform infrared spectroscopy (FTIR) spectra of pristine multi-walled carbon nanotubes (MWCNT).

**Figure 3:**
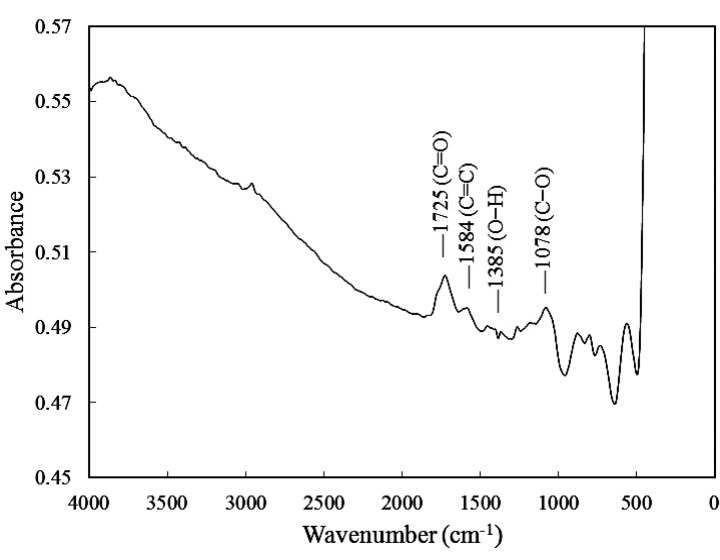
FTIR spectra of MWCNT-COOH.

Fourier transform infrared (FTIR) spectral analyses of the pristine MWCNT and MWCNT–COOH were performed to verify possible structural differences in the two series of samples. The evidence of chemical functionalization by oxidation on the surface of MWCNTs was conformed using Fourier transform infrared spectroscopy. FTIR spectra of MWCNT ground in KBr pellet were recorded on a Bomam DA 8.3 spectrometer, with 200 scans averaged at a resolution of 1 cm^−1^. Figure 2 and Figure 3 show the FTIR spectra of the pristine MWCNT and MWCNT-COOH, respectively. The characteristic response of the FTIR spectra for MWCNT-COOH exhibits three distinctive peaks (C=O, O–H, C–O) that are considered as a result of oxidation forming the COOH groups on the surface of MWCNTs. The absorption band at 1725 cm^−1^ is corresponding to C=O stretching of COOH, while the absorption bands at 1385 cm^−1^ and 1078 cm^−1^ are associated with O–H bending and C–O stretching, respectively. The absorption band at 1584 cm^−1^ is more likely from the C=C stretching mode of carbon nanotubes. Similar observations of the FTIR spectra for MWCNT-COOH were reported by Theodore *et al.* [29] and Wu *et al.* [30].

## 4. Free Vibration Test

**Figure 4:**
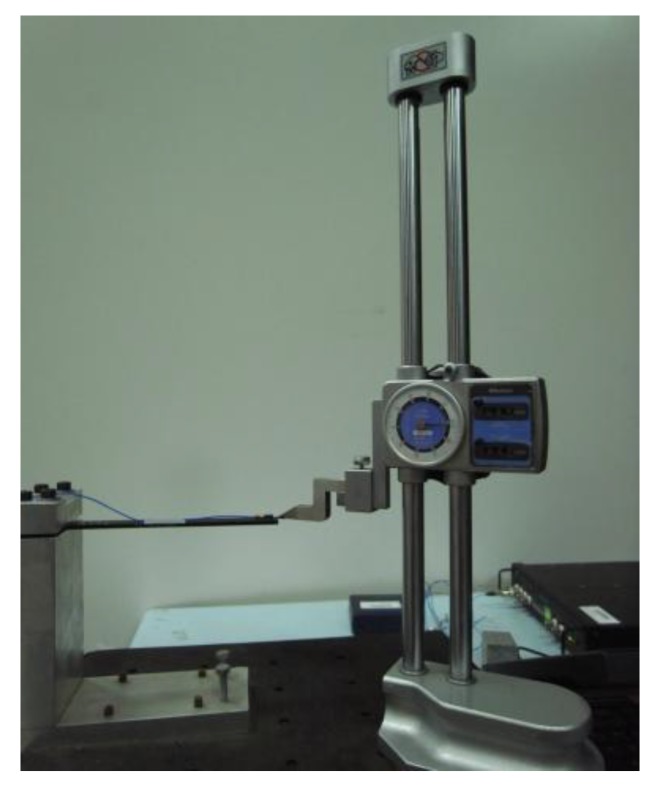
Experimental setup of free vibration test.

Damping and natural frequency are often used to characterize the dynamic behavior of structures undergoing various loading and boundary conditions. In order to determine the damping and natural frequency of the nanocomposites, free vibration tests of a cantilever beam were conducted to obtain the free decay behavior. The test specimen has a rectangular cross section of 15 × 3 mm^2^ and a length of 170 mm. The beam was clamped at one end to a much stiffer and heavier jig, using 20 mm of its length. An accelerometer weighting 1 g was placed near the free tip to measure the free vibration response of the cantilever beam. The experimental setup is shown in Figure 4.

**Figure 5:**
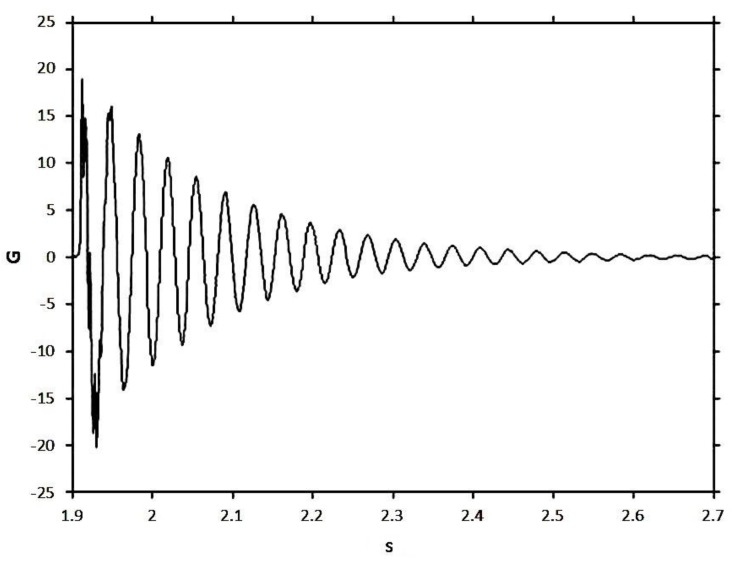
Time response of nanocomposite with 0.5 wt % of MWCNT-COOH in the free vibration test.

**Figure 6:**
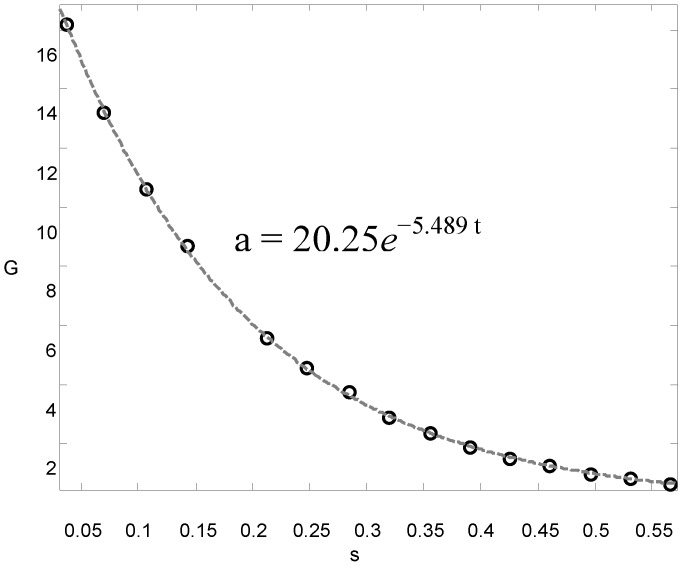
Amplitude decay of free vibration.

**Figure 7:**
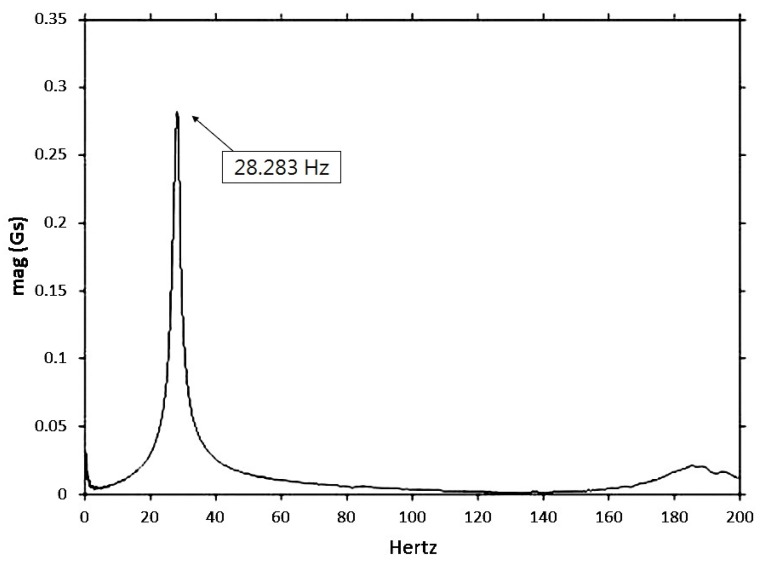
Frequency response of nanocomposite with 0.5 wt % of MWCNT-COOH in the free vibration test.

Displacing the free end by 10 mm and then releasing it excited the cantilever beam. This way, the fundamental mode was predominantly excited. The initial condition was introduced so that the displacement on the free end of the specimen was high enough to produce a good signal (high signal-to-noise ratio) but still within the linear behavior range. The MWCNT/epoxy and MWCNT-COOH/epoxy nanocomposites with different contents of MWCNTs ranging from 0.3 to 1.0 wt % were considered in this study to investigate the effects of functionalization and MWCNT on the damping ratio and natural frequency. A typical response of acceleration in the time domain for the cantilever beam with MWCNT-COOH addition 0.5 wt % is shown in Figure 5. It exhibits an oscillatory decay due to the damping. The decaying amplitude of the free vibration is plotted in Figure 6. The frequency response is obtained by performing Fast Fourier Transform (FFT) as shown in Figure 7. It can be observed that the beam vibrates predominantly at its fundamental mode with damped natural frequency of 28.125 Hz.

Basing on the theory of vibration, the free decay behavior of the acceleration for a single degree of freedom system with damping can be written as:

(1) $$a(t)=Ae^{-\varsigma \;\omega_{n}\;t}\cos(\omega_{d}t-\psi )$$

(2) $$\omega_{d}=\omega_{n}(1-\varsigma^{{}_{2}})$$

Where *ω_n_*, *ς* and *ψ* are the natural frequency, damping ratio and phase angle, respectively; *ω_d_* denotes the damped natural frequency.

Substituting the experimental results of free vibration amplitude (as shown in Figure 6) and damped natural frequency 28.283 Hz (as shown in Figure 7) into Equations (1) and (2), yield to:

(3) $$Ae^{-\varsigma \;\omega_{n}\;t}=20.25e^{-5.489\;t}$$

(4) $$\omega_{d}=2\pi f=177.71=\omega_{n}(1-\varsigma^{{}_{2}})$$

Utilizing Equations (3) and (4), the natural frequency *ω_n_* and damping ratio *ς* of the nanocomposite beam are readily to be determined.

Following the procedures as described above, the damping ratio and natural frequency of the nanocomposites with different additions of pristine MWCNTs and functionalized MWCNTs are able to be evaluated by the free vibration test. The data presented in this work are the average of three test specimens for each case and the deviation is less than 5%. The experimental results are shown in Table 1. It can be observed that the natural frequency of the nanocomposite is increasing with the increase of MWCNTs addition, while the damping ratio is decreasing with the increase of the MWCNTs addition. The natural frequency of MWCNT-COOH/epoxy is slightly higher than that of MWCNT/epoxy. The adhesion between the carbon nanotube and epoxy matrix is one of the most important factors that affects the stiffness and damping of the nanocomposites. Functionalized MWCNTs improved the interfacial bonding between the nanotubes and epoxy resin. The stiffness of the MWCNT-COOH/epoxy nanocomposite increases as a result of good adhesion and better load transfer. Higher stiffness leads to a higher natural frequency of the MWCNT-COOH/epoxy comparing with MWCNT/epoxy. Good adhesion between the MWCNT-COOH and epoxy yields to less slippage at the interface which will result in a less dissipation of energy and a smaller damping ratio.

**Table 1:** Natural frequency and damping ratio of MWCNT/epoxy and MWCNT-COOH/epoxy.

wt %	MWCNT/epoxy			MWCNT-COOH/epoxy	
	Natural frequency	Damping ratio		Natural frequency	Damping ratio
0	26.94 Hz	0.03647		26.94 Hz	0.03647
0.3	27.75 Hz	0.03282		28.25 Hz	0.03260
0.5	28.06 Hz	0.03093		28.31 Hz	0.03086
0.8	28.75 Hz	0.03062		29.31 Hz	0.03015
1.0	29 Hz	0.02968		29.94 Hz	0.02933

## 5. Conclusions

The effect of MWCNTs on the structural dynamic behavior of nanocomposites was investigated using free vibration test. Two different types of MWCNTs, pristine MWCNTs and functionalized MWCNTs, were used in this study. MWCNTs were functionalized through oxidation. Carboxylic acid groups were grafted onto the surface of MWCNTs via sonication in sulfuric-nitric acid. The covalent linkage between the carbon nanotube and functional group was characterized by FTIR. The attachment of functional groups onto MWCNTs significantly improves their solubility and promotes the homogeneous dispersion of MWCNTs in solvents as well as strong interfacial adhesion between the MWCNTs and epoxy matrix. The natural frequency of the nanocomposites is increasing with the increase of MWCNT loading while the damping ratio is decreasing with the increase of MWCNT. Good adhesion between the MWCNT-COOH and epoxy leads to higher stiffness and less slippage at the interface which will result in a higher natural frequency and smaller damping ratio.
